# Assessment of anxiety and depression among hospitalized COVID-19 patients in Thailand during the first wave of the pandemic: a cross-sectional study

**DOI:** 10.1186/s41983-021-00362-9

**Published:** 2021-08-03

**Authors:** Wajana Thaweerat, Wannarat Amornnimit Pongpirul, Wisit Prasithsirikul

**Affiliations:** 1 Division of Radiation Oncology, Department of Radiology, Faculty of Medicine Siriraj Hospital, Mahidol University, Bangkok, Thailand; 2 Bamrasnaradura Infectious Diseases Institute, Department of Disease Control, Ministry of Public Health, Nonthaburi, Thailand

**Keywords:** Anxiety, COVID-19, Depression, Psychological tests

## Abstract

Anxiety and depression in hospitalized COVID-19 patients in Thailand during the first wave of the pandemic were investigated. Thai version of Hospital Anxiety and Depression Scale (HADS) was chosen as an instrument for evaluation. Thirty-two voluntary participants completed the questionnaire. Three (9.4%) respondents had abnormal anxiety sub-scale scores while no respondents had abnormal depression sub-scale scores. There was no statistical demographic difference between the anxiety and non-anxiety groups.

## To the editor,

## Introduction

COVID-19 patients have been identified as vulnerable groups that might develop serious mental health consequences [1]. Uncertain clinical progression by a novel pathogen and patient isolation might lead to psychological distress in patients [2]. Hence, our study aims to evaluate the mental health of these patients.

## Main text

Fifty-two hospitalized PCR-confirmed non-severe COVID-19 patients older than 18 years admitted at Bamrasnaradura Infectious Disease Institute in April 2020 during the first wave of pandemic were invited to participate. The Hospital Anxiety and Depression Scale (HADS) [3] was used to measure anxiety and depression. It consisted of a 7-item anxiety sub-scale (HADS-A) and a 7-item depression sub-scale (HADS-D). HADS was translated in Thai and had been validated in hospitalized patients [4]. The questionnaire was completed within the first 5 days of admission and was analyzed by the physician. Demographic data were reported by descriptive statistics. Two-tailed Fisher’s exact test and two-tailed *t*-test were used to compare categorical variables and continuous variables, respectively. All statistical analyses were performed in Microsoft Excel 2013.

Thirty-two hospitalized COVID-19 patients voluntarily completed the questionnaire. The mean ± standard deviation (SD) of HADS-A score was 6.7 ± 3.7 while the mean ± SD of HADS-D score was 4.1 ± 2.8. When using the cut-off scores at 11, three participants (9.4%) had abnormal HADS-A scores while no participants had abnormal HADS-D scores. Participants with abnormal HADS-A score were properly evaluated by a psychologist. Further analysis revealed no statistical difference in demographic characteristics between anxious and non-anxious participants (Table 1).

**Table 1:** Characteristics comparison between anxiety and non-anxiety participants

Characteristics	Anxiety group (*n* = 3)	Non-anxiety group (*n* = 29)	*p*-value
Gender			
Male	0 (0%)	14 (48.3%)	0.23
Female	3 (100%)	15 (51.7%)	
Age (year; mean ± SD)	45.3 ± 22.0	36.4 ± 15.1	0.39
Educational background			
Secondary school level or lower	1 (33.3%)	14 (48.3%)	1.00
University level	2 (66.7%)	15 (51.7%)	
Place of origin			
Bangkok Metropolitan Region	1 (33.3%)	20 (69.0%)	0.27
Other regions	2 (66.7%)	9 (31.0%)	
Income (Baht; mean ± SD)	20,333 ± 13,650	29,776 ± 34,363	0.39
Underlying diseases			
Yes	2 (66.7%)	9 (31.0%)	0.27
No	1 (33.3%)	20 (69.0%)	

*SD* standard deviation

From the literature review, several studies assess anxiety and depression in hospitalized COVID-19 patients by using HADS. Three hospitals in China revealed that the prevalence of admitted COVID-19 patients with abnormal HADS-A score is 20.9–41.5% while the prevalence of patients with abnormal HADS-D score is 18.6–50.5% [5–7]. Two studies from South Korea conducted in hospitalized COVID-19 patients showed that 10.3–18% of participants had abnormal HADS-A score, whereas 15.9–39% of participants had abnormal HADS-D score [8, 9]. Furthermore, an evaluation of anxiety and depression among admitted COVID-19 patients in Turkey demonstrated that 34.9% and 42.0% of participants had abnormal HADS-A score and abnormal HADS-D score, respectively [10].

The biological effect of SARS-CoV-2 on the brain causing neuropsychiatric symptoms is not well-established, but possible mechanisms include neuroinflammation, altered neurotransmitter, and neuronal damage [11]. For psychosocial stressors, the uncertainty of disease progression, welfare concern of quarantined contact people who might be infected, and an obstacle of mental support from visitors in the isolation ward can contribute to the development of anxiety in these vulnerable patients. Nevertheless, our study is limited by a small sample size due to the decline of patients during the duration of our study.

## Conclusions

In summary, we suggested that mental health assessment is crucial for the hospitalized COVID-19 patient to explore psychological distress which might require intervention to prevent further psychological complications. Our study and other studies conducted overseas demonstrated that HADS can be used to assess depression and anxiety in hospitalized COVID-19 patients.

## Data Availability

The datasets used and analyzed during the current study are available from the corresponding author on reasonable request.

## Abbreviations

HADS: Hospital Anxiety and Depression Scale
HADS-A: Anxiety sub-scale of HADS
HADS-D: Depression sub-scale of HADS
SD: Standard deviation
